# Wnt/β-catenin pathway activation is associated with glucocorticoid secretion in adrenocortical carcinoma, but not directly with immune cell infiltration

**DOI:** 10.3389/fendo.2025.1502117

**Published:** 2025-03-10

**Authors:** Tanja Maier, Laura-Sophie Landwehr, Alexandra Triebig, Stefan Kircher, Marc P. Schauer, Thomas Knösel, Silviu Sbiera, Paul Schwarzlmueller, Petra Zimmermann, Martin Reincke, Isabel Weigand, Martin Fassnacht, Matthias Kroiss

**Affiliations:** ^1^ Department of Medicine IV, Ludwig-Maximilians-Universität (LMU) University Hospital, LMU Munich, Munich, Germany; ^2^ Department of Internal Medicine I, Division of Endocrinology and Diabetes, University Hospital, University Würzburg, Würzburg, Germany; ^3^ Institute of Pathology, University of Würzburg, Würzburg, Germany; ^4^ Institute of Pathology, Ludwig-Maximilians-Universität (LMU) Munich, Munich, Germany; ^5^ Department of General, Visceral and Transplant Surgery, University Hospital, Ludwig-Maximilians-Universität (LMU) Munich, Munich, Germany; ^6^ Comprehensive Cancer Center Mainfranken, University of Würzburg, Würzburg, Germany

**Keywords:** immunohistochemistry, LEF-1, hypercortisolism, prognosis, marker, immune cell infiltration, glucocorticoid receptor, adrenocortical carcinoma

## Abstract

**Background:**

In advanced adrenocortical carcinoma (ACC), the response rate to immune checkpoint inhibition (ICI) is only ~15%. Glucocorticoid (GC) secretion and the activation of the Wnt/β-catenin pathway have been suggested to contribute to low tumour immune cell infiltration. The transcription factor lymphoid enhancer factor 1 (LEF-1) transduces β-catenin (*CTNNB1*)-mediated transcriptional activation.

**Objective:**

To understand the contribution of Wnt/β-catenin pathway activation and glucocorticoid receptor (GR) signalling to the immunologically cold ACC tumour microenvironment.

**Methods:**

Semi-quantitative immunohistochemistry (IHC) of β-catenin (CTNNB1), LEF-1, GR and T cell markers CD3, CD4, CD8, Fox P3 in 59 ACC samples. Targeted RNA expression analysis of 354 immune-related genes in 58 additional ACC tissue specimens. Correlative analyses with clinical data.

**Results:**

Nuclear LEF-1 and CTNNB1 protein expression were positively correlated in ACC tissue (Pearson R^2^ = 0.1283, p=0.0046). High, moderate and low protein expression was detected in 24.1%, 53.2% and 19.3% of samples for LEF-1, and 30.6%, 43.5% and 19.3% for CTNNB1, respectively. We found higher LEF-1 expression in GC-secreting tumours which did not differ from inactive tumours in terms of GR expression. T cell markers, as evaluated by IHC, were not associated with expression of Wnt/β-catenin pathway markers. At RNA level, tumours with high LEF-1 expression showed significant downregulation of 37 transcripts (including 8 involved in antigen presentation). High LEF-1 expression levels correlated with worse overall survival in this cohort. This was not the case for CTNNB1 and GR.

**Conclusion:**

Lef-1 expression is useful as a biomarker of activated Wnt/β-catenin signalling in ACC. Wnt/β-catenin pathway activation was not associated with reduced immune cell markers in ACC but GC secretion and may be related to tumoural antigen presentation.

## Introduction

Adrenocortical carcinoma (ACC) is a rare and aggressive malignancy with an incidence of 0.6-2 inhabitants per million per year (1). Around 60% of patients show steroid hormone excess, which has been associated with poor outcome (2, 3).

The treatment options of advanced ACC are unsatisfactory. After resection with microscopically unaffected margins (R0), 50-80% of patients develop recurrent or metastatic disease (4–7). Mitotane is the only approved treatment for patients to control tumour growth and hypercortisolism. With a response rate of - at best - ~20% in carefully selected cases, mitotane monotherapy is of limited efficacy (8). Even in combination with cytotoxic chemotherapy, the response rate of only ~25% underlines the unmet medical need for novel treatment alternatives (9–11)

Immune checkpoint inhibitors (ICI) have revolutionised the treatment of many cancer entities but, unfortunately, not of ACC. The results of five clinical trials using different ICIs are heterogeneous and overall disappointing with objective response in only 17 (14%) of 121 treated patients (8, 12–16). A similar response rate was confirmed in a large retrospective analysis and a recent meta-analysis (17, 18). In none of these trials, established response markers to ICI such as tumour mutational burden or microsatellite instability were associated with response.

The tumour microenvironment of ACC has been reported to be depleted of T lymphocytes giving rise to the notion of ACC as an immunologically “cold” tumour entity (13), which is considered to contribute to the impaired responsiveness of ACC to immunotherapy.

Recently, our group has used clinical data of steroid hormone secretion together with IHC for T cell markers in 146 ACC tumours and found an immunosuppressive effect of clinical GC excess on T cell infiltration (13).

The Wnt/β-catenin pathway has been suggested as an additional cause for an immunologically silent tumour microenvironment in several types of cancer (19, 20). Activation of the Wnt/β-catenin pathway is a common driver event in ACC and caused by mutations in *CTNNB1* itself or by mutations in the upstream pathway gene *ZNRF3 (* 21, 22).

**Figure 1:**
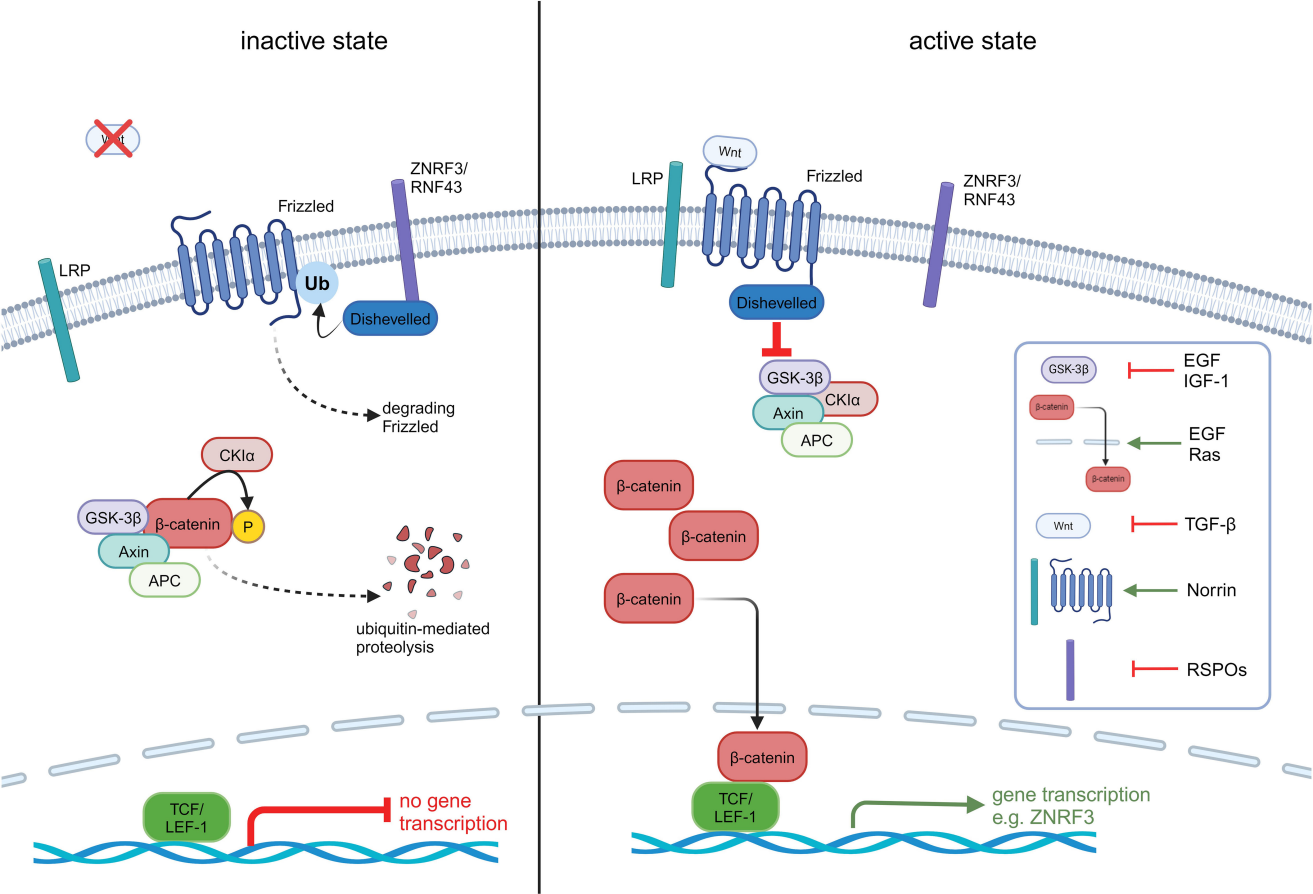
Overview of the inactive and active Wnt/β-catenin pathway in ACC. In the inactive state, β-catenin is bound to its destruction complex consisting of GSK3β, APC, Axin and CKIα. This complex leads to the ubiquitinylation and phosphorylation of β-catenin and its subsequent destruction. Is the pathway activated, the Wnt ligand binds to the receptor frizzled (FZD) which leads to an activation of dishevelled (DVL) and inhibits the destruction complex. As a consequence, β-catenin is protected from degradation, accumulates in the cytoplasm and translocates to the nucleus. In the nucleus, β-catenin binds to the N terminus of TCF/LEF-1 transcription factor and recruits co-factors to activate the transcription of Wnt target genes like LRP receptors through inhibition of E3 ubiquitin ligases ZNRF3. ZNRF3/RNF43 translocate to the plasma membrane and lead to an ubiquitinylation of FZD through DVL and degradation of FZD. This step leads to an inactivation of the Wnt/β-catenin pathway. Tumour cells need to overcome this strong negative feedback loop by mutating ZNRF3, appearing in 21% of ACC patients or mutating β-catenin (CTNNB1) appearing in 16% of patients (21, 22). In addition to Wnt ligands, various other molecules are capable of activating the Wnt/β-catenin pathway through diverse mechanisms. Growth factors like insulin-like growth factor 1 (IGF-1) activate the pathway via cross-talk with downstream signalling cascades. Norrin is known to activate the canonical Wnt/β-catenin pathway by binding to Frizzled-4 receptor and LRP5/6 co-receptor, similar to Wnt ligands. R-spondins (RSPOs) enhance Wnt signalling by stabilizing Frizzled and ZNRF3, RNF43 (24). These examples represent only a subset of the non-Wnt activators.

Established roles of canonical Wnt/β-catenin signalling include proliferation, apoptosis, migration and tissue homeostasis (14). The key steps and mechanisms of Wnt/β-catenin activation in ACC have recently been reviewed elsewhere (23) and are depicted in **Figure 1**. In the final step of this pathway, CTNNB1 binds to the transcription factors TCF/LEF-1 (25) and triggers expression of target genes like c-Myc, cyclin D1 and CDKN1A (26).

Beyond its classical pro-tumourigenic functions, Wnt/β-catenin activation has been found to be associated with T cell-depletion across tumour entities including ACC (27, 28).

Mechanistically, Wnt signalling regulates peripheral T cell activation and differentiation of CD4^+^ T helper and CD8^+^ cytotoxic T cells (CTLs) (29). In melanoma cell lines as well as dendritic cells (DC), experimental overexpression of CTNNB1 has been shown to increase interleukin (IL) IL-10 secretion and to impede the ability of DCs to cross-prime tumour-targeting CD8^+^ T cells (30). Cancer cells secrete Wnt ligands that interact with frizzled (FZD) receptors on DCs, thereby inhibiting the production of IL-12 and promoting the differentiation of regulatory T cells (T_regs_) (31, 32).

Conversely, the production of IL-1β by tumour-associated macrophages has been shown to result in the phosphorylation and the subsequent stabilisation of β-catenin in colon cancer (33).

Despite the prominent role of this pathway in ACC, a systematic study of Wnt/β-catenin signalling and its potential interconnections with tumour-infiltrating immune cells has not been conducted so far.

Therefore here, we aimed to elucidate (i) the impact of Wnt/β-catenin pathway activation on the number of tumour-infiltrating T cells, (ii) the relationship between Wnt/β-catenin pathway activation and GC signalling and (iii) the immune-related mechanisms affected by Wnt/β-catenin signalling in the tumour microenvironment. To this end, we used IHC to assess LEF-1, CTNNB1 and GR expression and applied mRNA gene expression analysis of immune-related transcripts in clinically annotated ACC tissue samples.

## Patients and methods

### Tissue samples and clinical data

Tissue samples and clinical data from ACC patients were obtained as part of the European Network for the Study of Adrenal Tumors (ENSAT) registry study that has been approved by the ethics committees of both participating institutions (approval numbers 379/10 LMU and 88/11 JMU). The study was conducted in accordance with the principles of the Declaration of Helsinki and all patients provided written informed consent.

Anonymised colon mucosa samples (N=14) and normal adrenal glands (N=15) obtained from patients treated for renal carcinoma served as controls. Data of participating patients are summarised in **Table 1** and **Supplementary Table S1**.

**Table 1:** Clinical characteristics of patients and tumours categorised according to LEF-1 (median 48.0) and CTNNB1 RNA expression (median 49.5) based on Nanostring nCounter.

	β-catenin low	β-catenin high	Whole cohort
Patients n	28	30	58
Age at diagnosis (range)	51.5 (30-77)	49.5 (18-74)	48 (18-77)
Sex n (%)			
Female	14 (50%)	23 (77%)	37 (64%)
Male	14 (50%)	7 (23%)	21 (36%)
ENSAT stage (I-IV)			
I	0	2 (7%)	2 (3%)
II	12 (43%)	6 (20%)	18 (31%)
III	6 (21%)	8 (27%)	13 (22%)
IV	10 (36%)	11 (36%)	19 (33%)
unknown	0	3 (10%)	6 (10%)
Hormone status			
Inactive	10	4	14
GC	4	11	15
A	9	7	16
GCA	6	7	13
R status (ENSAT I-III)			
0	20 (71%)	19 (63%)	39 (67%)
1	7 (25%)	3 (10%)	7 (12%)
2	0	1 (3%)	1 (2%)
unknown	1 (4%)	7 (24%)	11 (19%)
Ki-67 index n (%)			
<10	8 (28%)	4 (13%)	12 (21%)
≥10	17 (61%)	24 (80%)	41 (70%)
unknown	3 (11%)	2 (7%)	5 (9%)
Tumour diameter (range in cm)	10 (3-22)	10 (5-18)	10 (3-22)

	LEF-1 low	LEF-1 high	Whole cohort
Patients N	32	26	
Age at diagnosis (range)	48 (32-77)	48 (18-74)	
Sex n (%)			
Female	15 (47%)	22 (87%)	
Male	17 (53%)	4 (13%)	
ENSAT stage (I-IV)			
I	1 (3%)	1 (4%)	
II	12 (38%)	6 (23%)	
III	6 (19%)	7 (27%)	
IV	8 (25%)	11 (42%)	
unknown	5 (15%)	1 (4%)	
Hormone status			
Inactive	11	3	
GC	6	9	
A	9	7	
GCA	3	10	
R status (ENSAT I-III)			
0	23 (72%)	16 (62%)	
1	3 (9%)	4 (15%)	
2	0	1 (4%)	
unknown	6 (19%)	5 (19%)	
Ki-67 index n (%)			
<10	10 (31%)	2 (8%)	
≥10	19 (60%)	23 (88%)	
unknown	3 (9%)	1 (4%)	
Tumour diameter (range in cm)	10 (3-17)	10 (5-22)	

### Immunohistochemistry

Full formalin-fixed paraffin-embedded (FFPE) tissue sections (3 µm) mounted on glass slides were deparaffinised in xylene and rehydrated in increasing dilutions of ethanol. An antigen retrieval step of 15 min with citric acid monohydrate (pH 6.5; Sigma Aldrich) in a high temperature vapour bath was performed and the endogenous peroxidase activity was quenched with 3% H_2_O_2_ (VWR International, USA) in methanol (Carl Roth) for 10 min at room temperature (RT). Subsequently, any possible unspecific antibody binding to active Fc receptors was blocked for 1 h at RT using 20% human type AB serum (Sigma Aldrich, Steinheim, Germany, Lot. 017K0443) in phosphate buffer saline (PBS). The slides were then incubated overnight at 4°C with anti-LEF-1 (1:75, Abcam, EPR2029Y), anti-β-catenin (1:2000. TD/BD Biosciences, clone 14) or anti-GR (1:100, Cell signalling, D6H2L) primary antibody (Novus Biologicals) and for negative control, with N-Universal Negative Control Rabbit antibody (Dako). The slides were then washed 5 times with PBS and the primary antibody signal was developed using the HiDef Detection Polymer System Detection Amplifier and HRP Polymer Detector (medac, 954D-20). After counterstaining with haematoxylin, the arrays were then analysed independently by two investigators and different staining intensities were scored semi-quantitatively calculated as followed: (1x percentage of weak staining) + (2x percentage of moderate staining) + (3x percentage of strong staining), ranging from 0 to 300 (34). Negative tissue samples were re-analysed in full tissue sections.

The cohort used for immunofluorescence and quantification of immune marker proteins was a sub cohort of the cohort from Landwehr et al. and staining was conducted as described previously (13). Briefly, the antibodies used were anti-CD3 (1:50, abcam; ab699), anti-CD4 (1:1000, abcam; ab133616), anti-CD8 (1:1000, abcam; ab4055) and anti-FoxP3 (1:40, abcam; ab20034); to investigate the correlation of LEF-1, CTNNB1 and GR expression data.

### NanoString nCounter^®^ gene expression analysis

To investigate the tumour microenvironment, NanoString nCounter^®^ (NanoString Technologies) was used according to manufacturer´s instructions. Total RNA was extracted by AllPrep DNA/RNA FFPE Kit (Qiagen) from two to five slides of 2-4 μm FFPE tissue for each sample. RNA quantity and quality were assessed using NanoDrop2000 (Thermo Fisher Scientific). For samples that reached the quality standards (A260/A280 ≥ 1.7 and A260/A230 ≥ 1.8), we evaluated the expression profile by NanoString using a customised profiling panel (NanoString Technologies) consisting of 354 GC- and immune--related genes plus eleven reference genes. Analysis of detected gene counts was performed using the nSolver Analysis Software 4.0 (NanoString Technologies). First, samples were selected by proving imaging quality controls according to: percentage of fields of view (FOV) read (>75%), binding density (between 0.05 and 2.25), positive control linearity (>0.95), and positive control limit of detection (>2). For high quality samples, raw gene counts were normalised to technical controls, reference genes and positive control (with the geometric mean as normalisation factor). Background subtraction was included in the advanced analysis settings. After completion of normalisation processes, counts were log2 transformed and a ratio analysis was performed by comparing the expression profiles of samples with high and low (cutoff: median) *LEF1* RNA expression. For each comparison, the p value (as one-tailed Student t test) and the false discovery rate (FDR) obtained using the Benjamini–Yekutieli method were calculated. Finally, genes were ranked based on fold change (FC; ≥ 2) and FDR (<0.1) setting. Bioinformatic analyses on gene expression profiling were conducted using R Software v3.3.2.

### Statistical analysis

Statistical analyses were performed using Prism (V.10.1.1, GraphPad Software Inc, La Jolla, California, USA) and SPSS (SPSS Statistics 29.0.0.0, IBM, Stanford University, California, USA). All statistically generated data are presented as median and range with p<0.05 considered statistically significant. For differences in H-scores, an unpaired non-parametric Mann-Whitney test with median values and 95% confidence interval (CI), was performed; whereas, for per sample analyses, a non-parametric Wilcoxon matched-pairs signed-rank test was used. For correlations between immune infiltration and staining intensity, a simple linear regression was used. Correlations between expression levels, mutational status and gender was performed using Fisher´s exact test.

## Results

### LEF-1 and CTNNB1 expression are correlated at protein level

**Figure 2:**
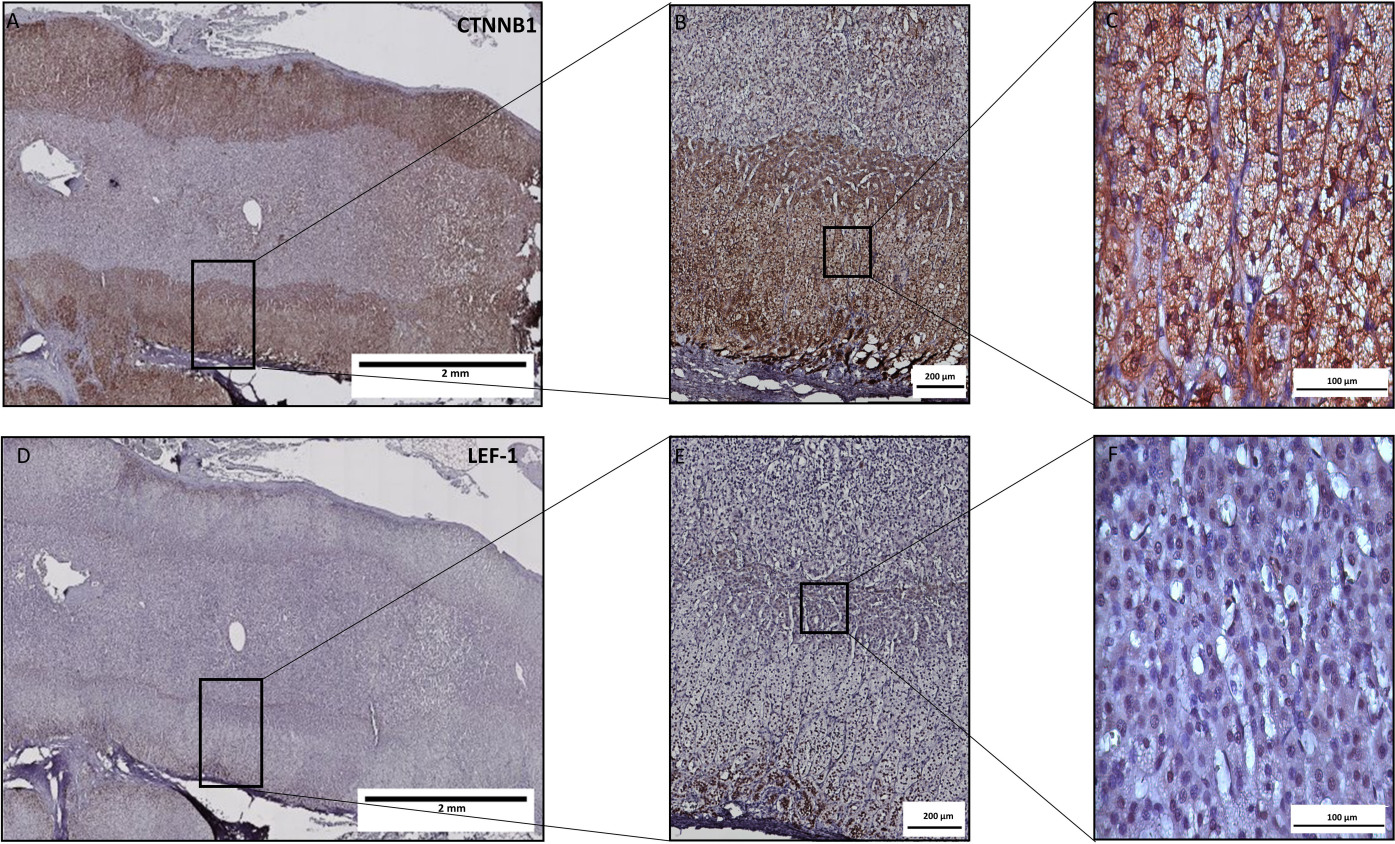
LEF-1 and CTNNB1 protein expression by immunohistochemistry in a healthy adrenal gland. Consecutive tissue slices were stained for LEF-1 **(A–C)** or CTNNB1 **(D–F)**. While LEF1 expression is clearly expressed predominantly in the subcapsular zone and zona glomerulosa with exclusive nuclear expression, CTNNB1 is more diffusely expressed with both nuclear and cytoplasmic staining.

To assess Wnt/β-catenin pathway activation in ACC tissue samples we used IHC. The clinical characteristics of ACC patients are provided in **Supplementary Table S1**. In addition to the semi-quantitative assessment of CTNNB1, we used the expression of the CTNNB1 target LEF-1 as a downstream indicator of canonical Wnt/β-catenin pathway activity. Analysis of CTNNB1 by IHC is hampered by its expression both in the cytoplasm and the nucleus (**Figure 2**) that leads to diffuse staining and uncertainty about the actual pathway activation. The specificity of the antibody staining was tested in healthy colon mucosa (N=14), where a specific localisation in crypts was confirmed in line with pathway activation in dividing mucosa cells (**Supplementary Figure S1**). In healthy adrenal tissue (N=15) (**Figure 2**), we found strong nuclear LEF-1 expression in the subcapsular zone and the *zona glomerulosa* in line with the current model of centripetal cell renewal originating from the subcapsular area in the human adrenal cortex (35).

**Figure 3:**
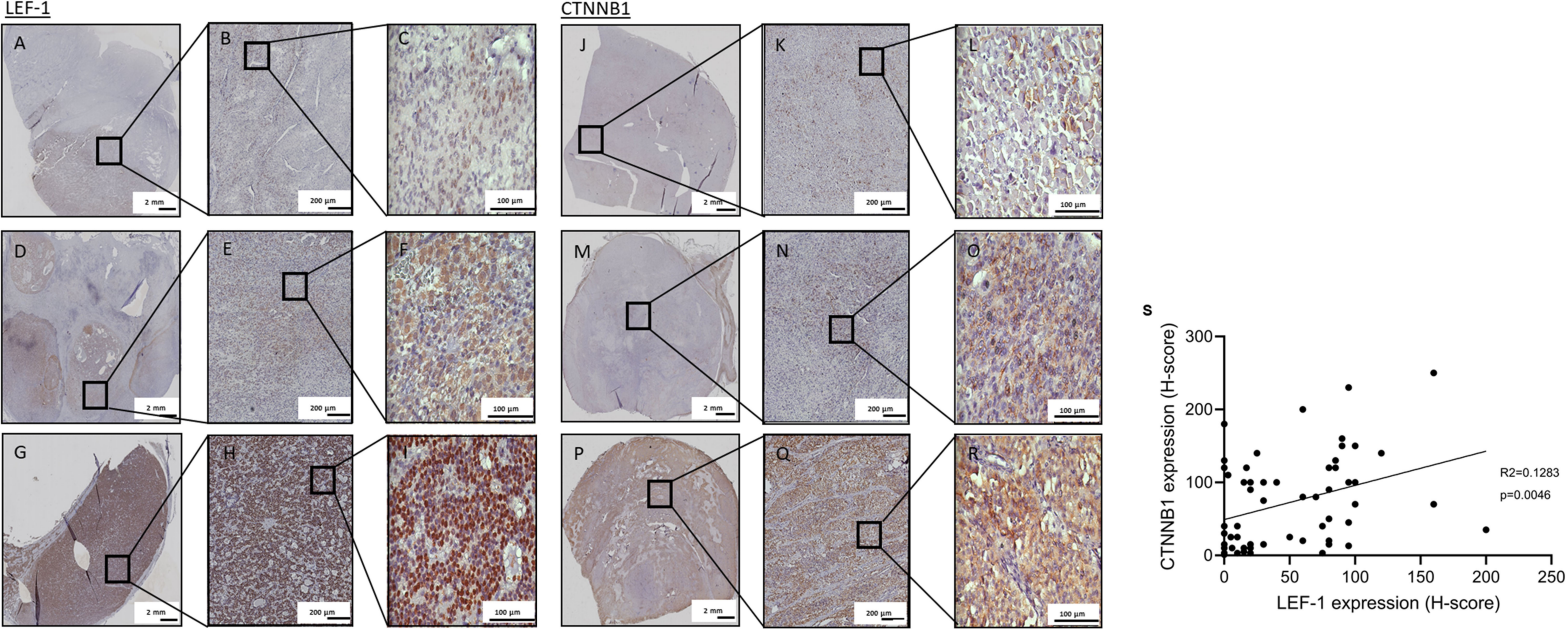
LEF-1 and CTNNB1 immunohistochemistry staining of full FFPE sections. Representative specimens of full ACC tissue sections stained for LEF-1 **(A–I)** or CTNNB1 **(J–R)** are shown with low **(A–C, J–L)** intermediate **(D–F, M–O)** and high **(G–I, P–R)** expression of LEF-1 and CTNNB1, respectively. Semi-quantitative assessment of expression was performed with H-score. **(S)** Correlation of semi-quantitatively assessed protein expression of nuclear LEF-1 and overall CTNNB1 (p value=0.0046).

Next, we investigated the expression of CTNNB1 and LEF-1 as potential markers of Wnt/β-catenin signalling in ACC. Based on the H-score, weak nuclear staining for LEF-1 was observed in 20 (35%), intermediate staining in 17 (29%) and strong staining in 21 (36%) out of 58 tumours. Representative examples are shown in **Figures 3A–I**. We semi-quantitatively scored CTNNB1 expression by considering both cytoplasmic and nuclear staining (36). Staining for CTNNB1 was weak in 19 (33%), intermediate in 16 (27%) and strong in 23 (40%) of 58 samples (**Figures 3J–R**). LEF-1 staining significantly correlated with CTNNB1 staining (R^2^= 0.1283, p= 0.0046), although with discrepancies at the individual sample level as these were not consecutive slides of the tumour (**Figure 3S**). Hence, we used LEF-1 expression as a marker for Wnt/β-catenin pathway activation.

### Correlation of Wnt/β-catenin pathway markers with immune cell infiltration

To assess whether the activation of the Wnt/β-catenin pathway is associated with tumoural immune infiltration, we compared LEF-1 and CTNNB1 expression between tertiles of cell numbers for CD3^+,^ CD4^+^, CD8^+^ and FoxP3^+^ T lymphocytes. For CD3^+^ T cells, low, intermediate and high immune infiltration corresponded to ≤2, 4 and ≥8.1 cells per high power field (HPF), respectively.

**Figure 4:**
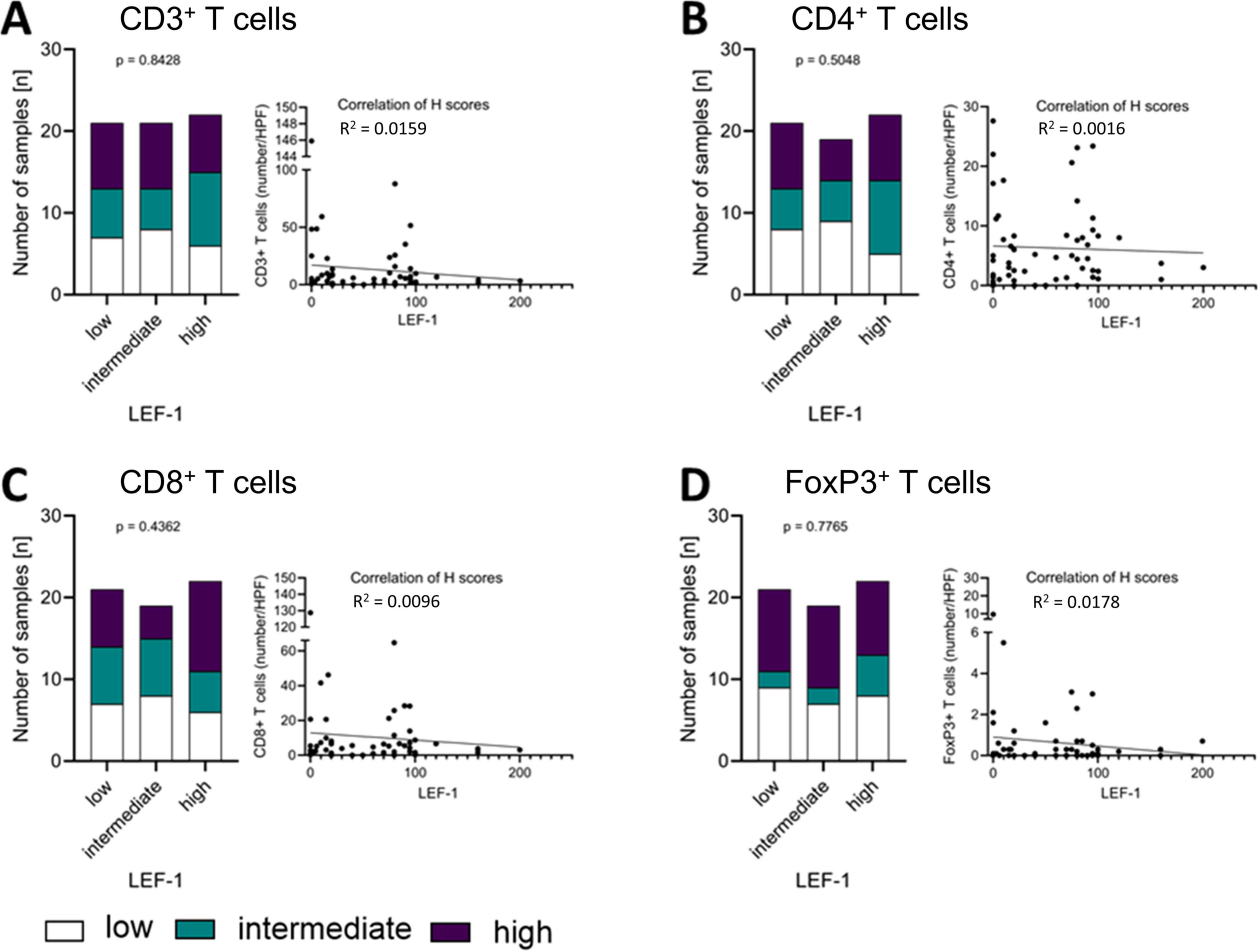
Correlation of LEF-1 and immune infiltration levels in the tumour. Protein expression levels of LEF-1 as well as CD3^+^ **(A)**, CD4^+^ **(B)**, CD8^+^ **(C)**, and FoxP3^+^ **(D)** levels (low: white; intermediate: green; high: purple) were divided into tertiles according to expression level and visualised in a stacked bar graph, respectively. Pearson correlation was used to show the linear relationship between the expression of LEF-1 (H-scores) and the immune cell infiltration (HPF), respectively. Testing for significance, fisher´s exact test was used.

Groups of T cell subtypes as measured by the numbers (**Supplementary Table S2**) of CD3^+^ (**Figure 4A**), CD4^+^ (**Figure 4B**), CD8^+^ (**Figure 4C**), and FoxP3^+^ T cells (**Figure 4D**) per HPF did not differ significantly between high, intermediate and low LEF-1 expression. Low numbers of CD3^+^ T cells in combination with low LEF-1 expression was present in 7 tumours. Intermediate infiltration by CD3^+^ T cells was present in 6 tumours. High infiltration by CD3^+^ T cells with high LEF-1 expression was present in 7 tumours. For comparison, 6 tumours had a high number of CD3^+^ T cells/HPF and intermediate LEF-1 expression. We did not observe any significant differences in the composition of T cell subtypes between tumours with low, intermediate and high LEF-1 expression (**Figures 4A–D**). When considering H-score and number of cells/HPF as continuous variables, the coefficient of determination (R^2^) was 0.0159 for CD3^+^, 0.0016 for CD4^+^, 0.0096 for CD8^+^, and 0.0178 for FoxP3^+^ T cells (all non-significant).

**Figure 5:**
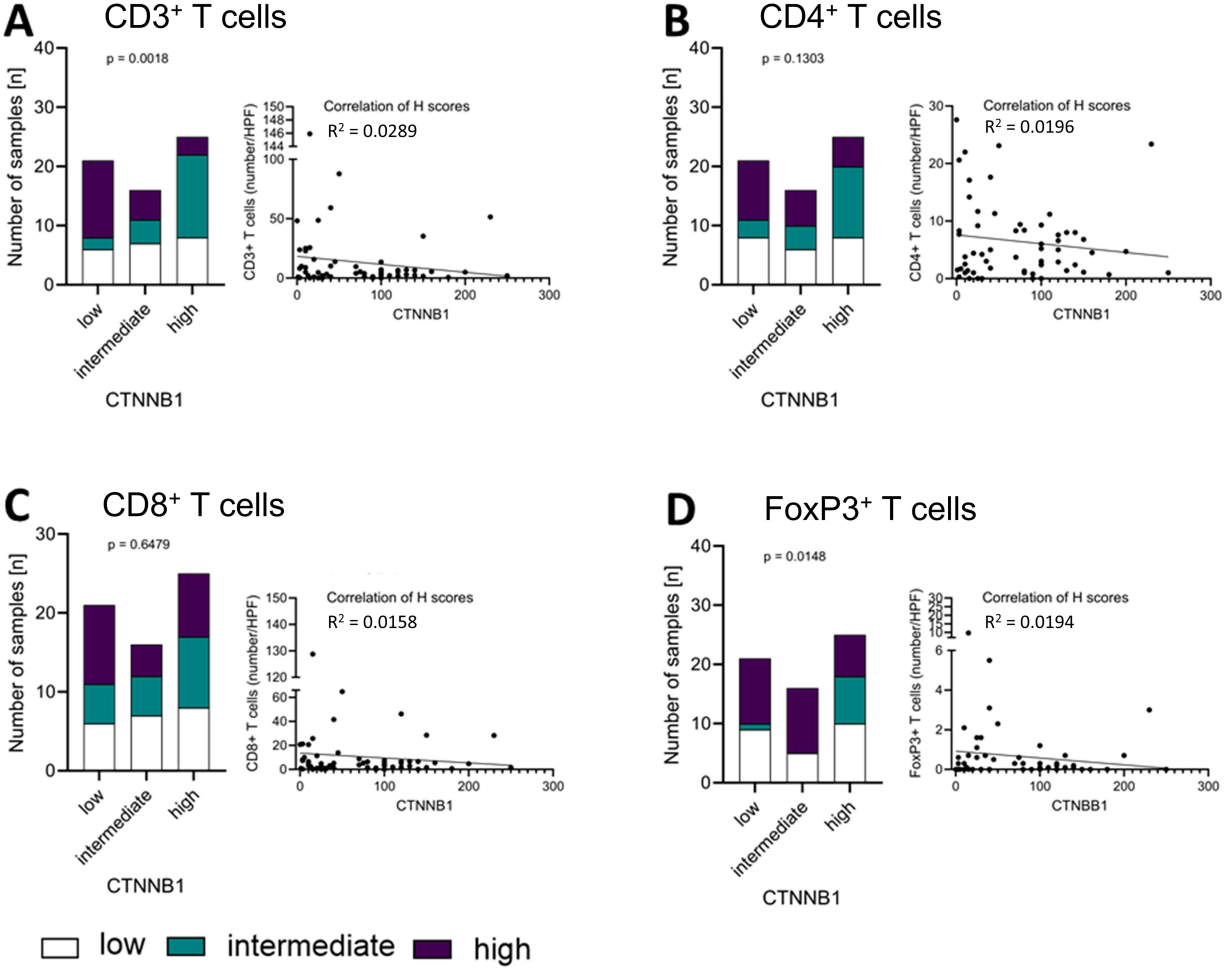
Correlation of CTNNB1 and immune infiltration levels in the tumour. Protein expression levels of CTNNB1 as well as CD3^+^ **(A)**, CD4^+^ **(B)**, CD8^+^ **(C)**, and FoxP3^+^ **(D)** levels (low: white; intermediate: green; high: purple) were divided into tertiles according to expression level and visualised in a stacked bar graph, respectively. Pearson correlation was used to show the linear relationship between the expression of CTNNB1 (H-scores) and the immune cell infiltration (HPF), respectively. Testing for significance, fisher´s exact test was used.

In contrast, we found statistically significant differences between groups of low, intermediate and high expression of CTNNB1 (**Supplementary Table S3**) regarding their CD3^+^ (p= 0.0018; **Figure 5A**) T cells, while we did not find it *vice versa*. However, in the analysis considering H-score and number of cells/HPF as continuous variables, R^2^ 0.0289 for CD3^+^, 0.0196 for CD4^+^, 0.0158 for CD8^+^, and 0.0194 for FoxP3^+^ T cells suggests that outliers with relatively high T cell infiltration were the drivers of the correlation of both markers. All other T cell markers did not show a significant correlation between CTNNB1 and CD4^+^ (p=0.1302; **Figure 5B**), CD8^+^ (p= 0.6479; **Figure 5C**) or FoxP3^+^ T cells (p= 0.1302; **Figure 5D**), respectively.

### Wnt/β-catenin pathway markers, steroid excess and survival

**Figure 6:**
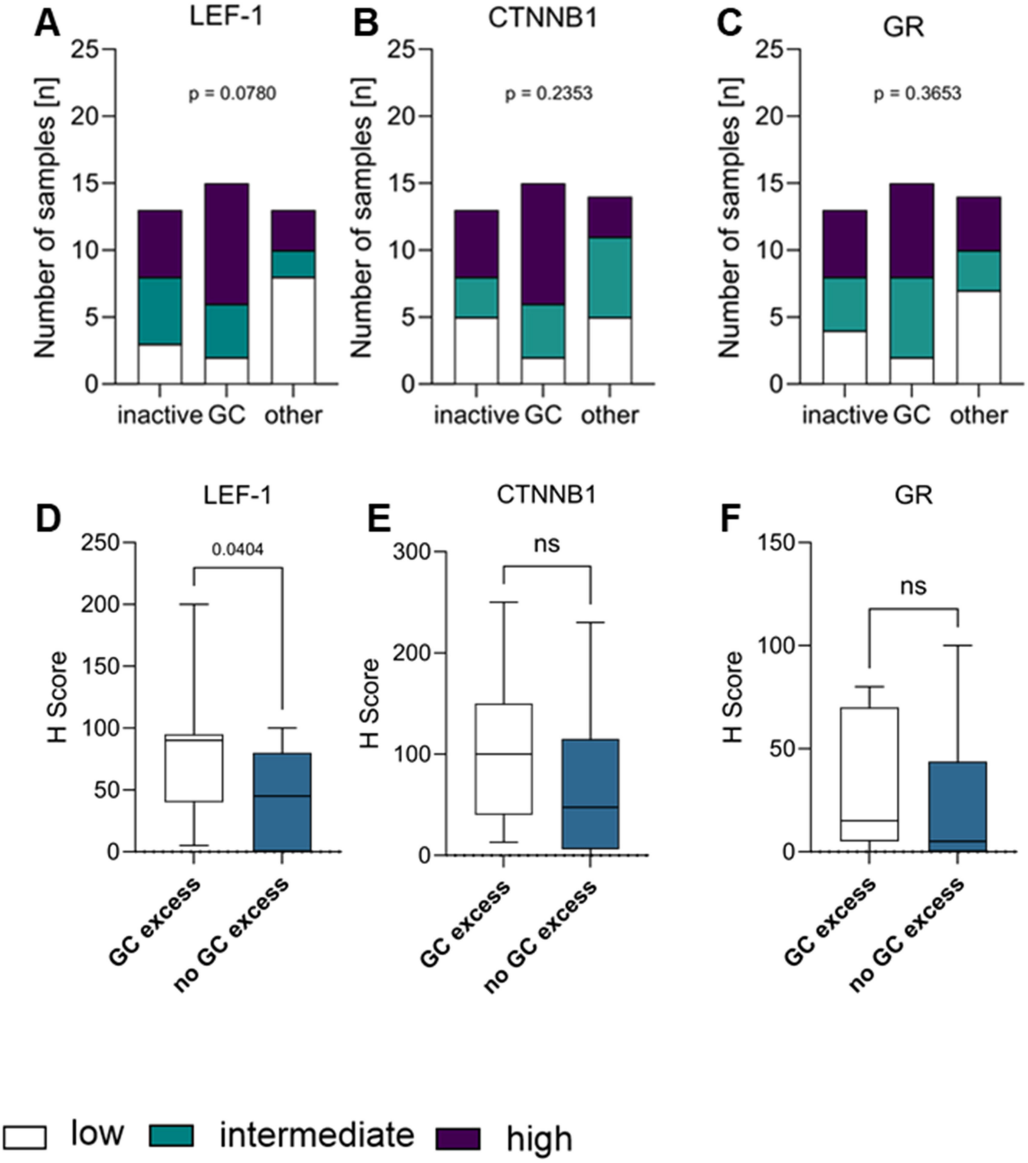
Correlation of LEF-1 **(A)**, CTNNB1 **(B)**, and GR **(C)** with the hormone status, respectively. Hormone status of tumour (inactive, glucocorticoid excess (GC) or other (GCA, A, GCAO) were correlated and visualised as a stacked bar graph correlating the hormone status with the protein expression levels of LEF-1 **(A)**, CTNNB1 **(B)** or GR **(C)**, respectively. H-score differences of LEF-1 **(D)**, CTNNB1 **(E)** and GR **(F)** between patients with GC versus hormonal inactive tumours combined with androgen excess tumours are shown in **(D–F)**. Statistical analysis was performed with fisher´s exact test.

When classifying tumours as secreting excess of GC only (GC, **Figure 6A**), GC together with androgens and precursors (GCA, **Supplementary Figure S3**) or being hormonally inactive, the proportion of GC-secreting tumours with strong LEF-1 expression (n=9/17) was higher compared to inactive tumours (n=5/17) or tumours with excess of multiple steroid hormones (n=3/17), however without statistical significance (p=0.0780). No difference was observed in terms of CTNNB1 staining intensity (**Figure 6B**) between groups.

Since tumoural NR3C1 expression has been proposed as a marker of GC excess in ACC (37), we compared protein GR expression between clinically GC-secreting and hormonally inactive tumours, but did not observe an association with clinical hormone status (**Figure 6C**, **Supplementary Figure S2**, **Supplementary Table S4**) suggesting that tumoural GR expression is not a marker of GC excess in ACC. In line, we did not observe an association of GR expression with either LEF-1 or CTNNB1 expression (**Supplementary Figure S4**).

We next interrogated clinical information on hormone excess that was available in 42 patients. When comparing all tumours with GC excess (n=15) to the remaining inactive and non-GC-secreting tumours (N=28), LEF-1 expression was significantly higher in GC-producing tumours (comprising GC and GCA) compared to non-GC-producing tumours with a median H-score of 70 (**Figure 6D**), but we did not observe a significant difference for CTNNB1 (**Figure 6E**) or GR (**Figure 6F**) expression in our dataset, respectively.

In other tumour entities it has been shown, that the abnormal activation of the Wnt/β-catenin pathway has an adverse prognostic impact (38–40). We, therefore, divided the samples into groups according to their expression of LEF-1 and CTNNB1 based on their median of the IHC H-score. We chose a dichotomous classification (high vs. low) with the median H-score as cut-off to account for the small number of patients for this analysis.

**Figure 7:**
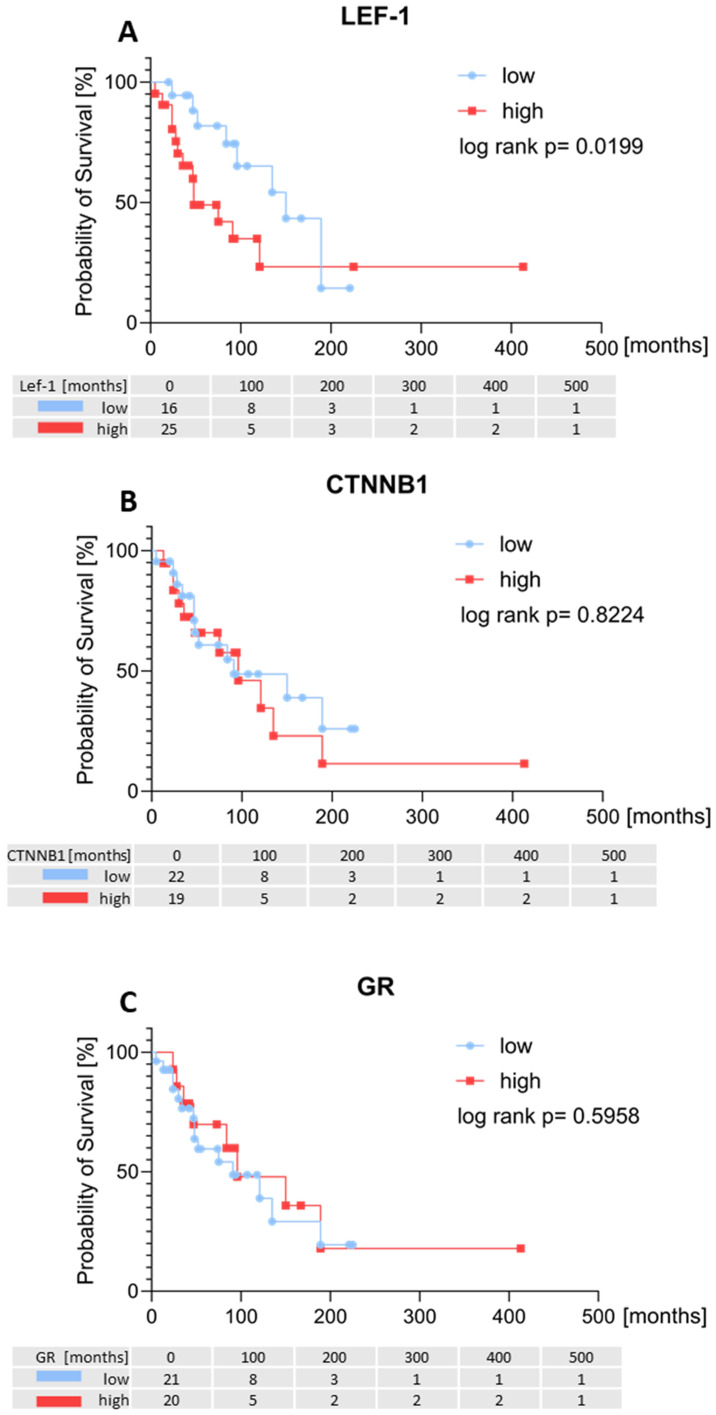
Overall survival in patients with adrenocortical carcinoma according to *LEF-1*, *CTNNB1* and *GR* determined by RNA analysis. Kaplan-Meier overall survival of all ACC patients with primary tumour samples (n=58) influenced by *LEF-1* **(A)**, *CTNNB1* **(B)** and *GR* **(C)** expression.

Patients with high tumoural LEF-1 expression had a significantly worse overall survival compared to patients with low LEF-1 expression (p=0.0199; **Figure 7A**). Patients with high LEF-1 expression had a significantly higher tumour stage at diagnosis (**Supplementary Figure S5**). In contrast, Kaplan-Meier survival analysis revealed no association of CTNNB1 or GR with the overall survival of the patients (**Figures 7B, C**).

### Expression of immune-related genes and activation of Wnt/β-catenin expression

**Figure 8:**
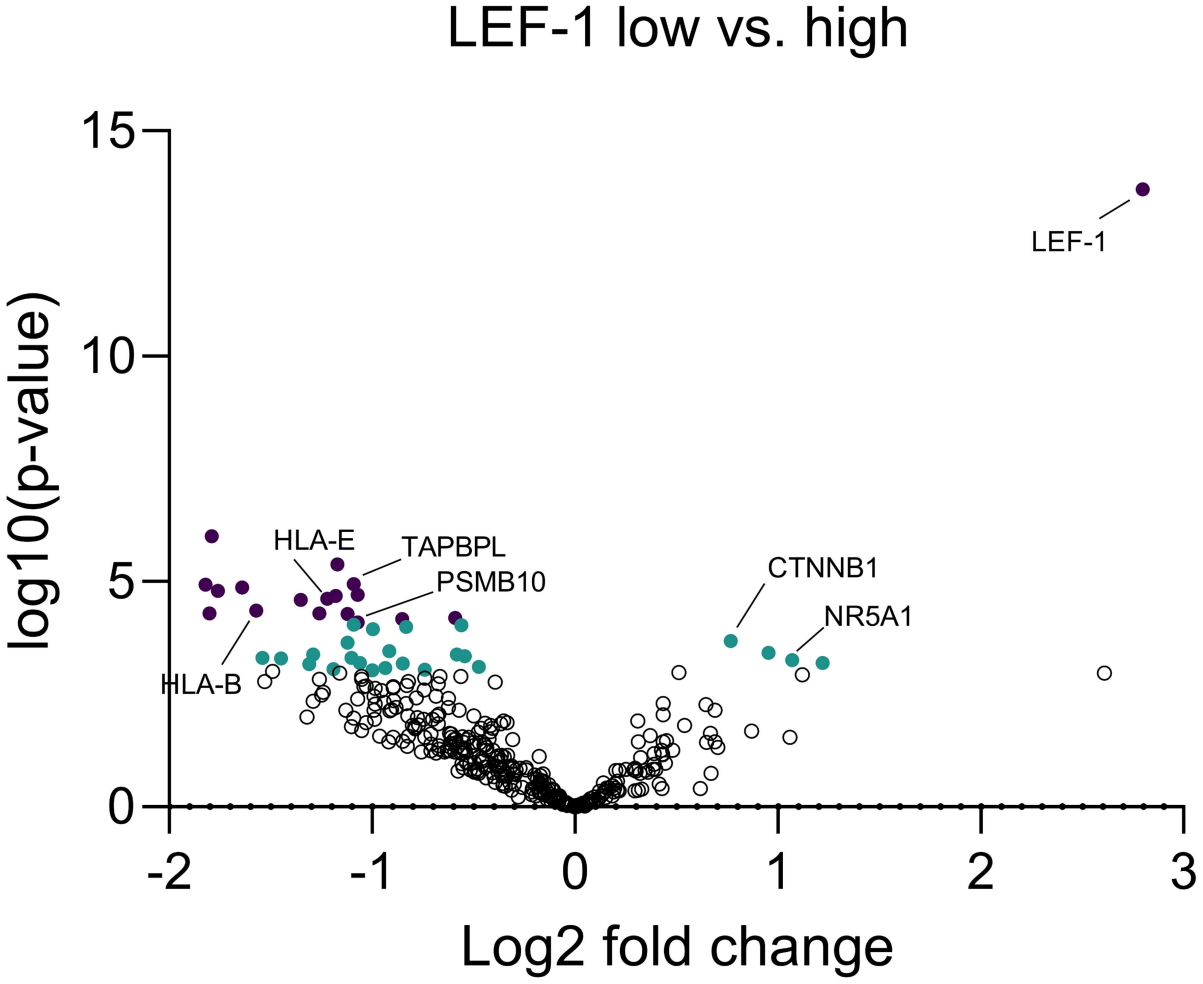
Expressional differences between LEF-1-high and LEF-1-low tumours at RNA level. Expression of 354 immune-related genes were measured and quantified by Nanostring nCounter® from FFPE tissue to analyse expressional differences between LEF-1-high and -low tumours. Significant genes (p<0.05-0.01) are marked in green and highly significant genes (p<0.01) are marked in purple. Analysis performed by Nanostring nSolver^®^ and Prism. Adjusted p value was calculated by Benjamini-Yekutieli.

Considering the significant impact of Wnt/β-catenin pathway activation on immune cell infiltration, we used a custom Nanostring nCounter^®^ panel of 354 genes to assess the expression of genes related to tumoural immunity and GC action (**Table 1**). When dividing the cohort according to median LEF-1 expression, we found significantly down-regulated expression of 37 genes in LEF-1^high^-expressing tumours (**Table 2**). These included TAPBPL, PSMB10 and CD27. Importantly, we found HLA-B, HLA-E, and PSMB10 all related to antigen presentation to be enriched among down-regulated genes in tumours with high LEF-1 expression (**Figure 8**, **Table 1**) suggesting an adverse impact of Wnt/β-catenin pathway on tumour recognition by immune cells.

**Table 2:** Statistically significant differences in genes compared between tumours with low and high LEF-1 expression in the cohort described in **Table 1** based on Nanostring nCounter^®^.

Gene	p-value	log10(p-value)	adjusted α
Lef1	1.94E-14	13.7121983	<0.001
PRKCA	9.73e-07	6.01188716	0.0011068
GIMAP6	4.13e-06	5.38404995	0.0031321
TAPBPL	1.15e-05	4.93930216	0.005119
HLA-DRB1	1.16e-05	4.93554201	0.005119
BIRC3	1.35e-05	4.86966623	0.005119
NKG7	1.63e-05	4.7878124	0.0052075
MMRN2	1.94e-05	4.71219827	0.0052075
STAT5A	2.06e-05	4.68613278	0.0052075
HLA-E	2.39e-05	4.6216021	0.0053155
PFKFB2	2.57e-05	4.59006688	0.0053155
HLA-B	4.4e-05	4.35654732	0.0078416
CCL5	5.07e-05	4.29499204	0.0078416
SYK	5.11e-05	4.2915791	0.0078416
GPR160	5.17e-05	4.28650946	0.0078416
RBL2	6.4e-05	4.19382003	0.0091005
HEBP1	6.85e-05	4.16430943	0.0091674
PSMB10	8.05e-05	4.09420412	0.0101748
GPSM3	9.08e-05	4.04191415	0.0106817
JAK1	9.39e-05	4.02733441	0.0106817
TSC22D3	0.000101	3.99567863	0.0109422
DLG3	0.000113	3.94692156	0.0116858
CTNNB1	0.000208	3.68193667	0.020575
IRF1	0.000226	3.64589156	0.021424
PER2	0.000348	3.45842076	0.0316697
NR1D1	0.000386	3.4134127	0.0337768
P2RY13	0.000415	3.3819519	0.033883
CRY1	0.000417	3.37986395	0.033883
PARP4	0.000455	3.3419886	0.0356959
LLGL2	0.00049	3.30980392	0.0362551
CD27	0.000494	3.30627305	0.0362551
PTGER4	0.000514	3.28903688	0.0365441
NR5A1	0.00056	3.25181197	0.0386081
MERTK	0.000641	3.19314197	0.0412596
PSMB9	0.000648	3.18842499	0.0412596
CD45RO	0.000665	3.17717835	0.0412596
HLA-DPB1	0.000671	3.17327748	0.0412596
FoxO3	0.000786	3.10457745	0.0470591
TLR4	0.00084	3.07572071	0.0490026
CSF3R	0.00089	3.05060999	0.0497818
NFKB1	0.000901	3.04527521	0.0497818
MMD	0.000919	3.03668449	0.0497818

The data have been publicly deposited (41). Significant genes (p<0.05-0.01) are marked in green and highly significant genes (p<0.01) are marked in purple.

It has already been shown that overexpression of FKBP5 RNA levels is associated with Cushing’s syndrome (42). By exploiting our Nanostring nCounter^®^ data, we did not find a significant association between FKBP5 expression levels and GC-related genes (**Supplementary Figure S6A**) or cortisol secretion, respectively (**Supplementary Figures S6B–D**).

## Discussion

Wnt/β-catenin pathway activation is a known driver event in solid tumours including ACC (43). Here, we aimed to determine whether the activation of this pathway is associated with reduced tumoural immune cell infiltration and to identify other potentially associated determinants of tumoural immunity, such as GC signalling.

We first established LEF-1 IHC as a marker of Wnt/β-catenin pathway activation in ACC and demonstrated LEF-1 expression to lack association with T cell infiltration. Surprisingly, LEF1 was higher expressed in GC secreting ACC and associated with shorter patient survival.

Wnt/β-catenin pathway activation signatures have been established from RNA expression data but are difficult to assess in clinical routine, especially when using FFPE samples.

We found LEF-1 expression to be positively correlated to CTNNB1 staining and an easy to assess marker of the Wnt/β-catenin pathway activation. In contrast, the actual expression of activated CTNNB1 is difficult to distinguish visually due to both cytoplasmic and nuclear staining which is particularly true in lipid-rich tumours like ACC. LEF-1 expression is exclusively nuclear and therefore can be readily assessed by IHC. LEF-1 expression may be used as a downstream readout of Wnt/β-catenin pathway activation. High levels of LEF-1 expression correlate with an active Wnt/β-catenin signalling. LEF-1 expression has been previously utilised as a marker in different tumour entities like acute lymphoblastic leukaemia, oral squamous cell carcinoma, and colorectal cancer to identify Wnt/β-catenin activated tumours (44, 45). To our knowledge, LEF-1 has not been studied in ACC.

Activating mutations in genes of the Wnt/β-catenin pathway occur in many solid tumour entities like colorectal cancer (46), melanoma (47) or lung cancer (48). Driver mutations comprise the *CTNNB1*, *ZNRF3*, *APC* or *Axin1* genes in most cases. To this date no mutations in LEF-1 have been discovered for any entity.

In ACC, Wnt/β-catenin pathway activation is frequent and occurs via activating mutations in the *CTNNB1* gene or inactivating mutations in *ZNRF3 (* 21, 49). It must be noted that for our study, mutational data of CTNNB1 and ZNRF3 were unavailable due to the retrospective nature of this study. Still, yet unidentified mechanisms may activate the Wnt/β-catenin pathway in ACC similar to other malignancies. As an example, hematopoietic malignancies drive transcriptional pathway activation through the downstream effector LEF-1 in a β-catenin independent manner (45, 50). Based on gene expression data, Assié et al. have demonstrated ACCs with an activating *CTNNB1* mutation to exhibit higher expression of LEF-1 compared to non-tumour adrenal samples and ACCs without *CTNNB1* mutation (21).

It has been shown in ovarian and colorectal cancer that Wnt/β-catenin pathway activation leads to significantly lower levels of mature CD8^+^ T cells. In colorectal cancer, higher β-catenin levels led to an inhibition of CCL4, a proinflammatory chemokine, and subsequently lower recruitment of CD103^+^ DCs thus impairing CD8^+^ T cell activation (27, 51, 52). In a pan-cancer study by Luke et al., ACC represented the tumour entity with strongest pathway activity which was in turn related to reduced T cell-driven inflammation (28). Using a gene signature approach to characterise tumoural immune cells, Thorsson et al. demonstrated *CTNNB1* mutations to be associated with the immune cell-depleted phenotype of ACC (53). Also in melanoma it was shown that T cell depleted tumours often have an activating CTNNB1 mutation (54). By using high LEF-1 levels as an indicator of Wnt/β-catenin pathway activation we did not observe this negative correlation in our ACC cohort. Previous work implying Wnt/β-catenin pathway activation in immune cell exclusion has relied on gene expression signatures and focused on the relationship between CTNNB1 expression and CD8^+^ T cell infiltration in adrenocortical carcinoma. Liu et al. used TCGA data to show an inverse correlation between CTNNB1 expression and CD8^+^ T cell infiltration in ACC (55). These findings mirror the generally low proportion of CD8^+^ cells in an independent set of ACC specimens observed by Landwehr et al. (13). The mechanism underlying this relationship is unclear. While our study may be too small to capture an association of CTNNB1 expression specifically with CD8^+^ T, we find the number of CD3^+^ T cells to be inversely associated CTNNB1 expression.

While it is established that Wnt/β-catenin activation is associated with poor prognosis in ACC, we observed lacking association between CTNNB1 nucleocytoplasmic staining and clinical parameters in our cohort. We therefore suggest that LEF-1 may represent a better marker of Wnt/β-catenin pathway activation in ACC. The activation of this pathway is closely related to cortisol excess associated with lesser T cell infiltration. It must be acknowledged that specifically the infiltration with CD8^+^ T cells may be induced by crosstalk of the Wnt/β-catenin pathway not transduced via LEF1. This phenomenon of signal crosstalk has been reviewed by Zhou et al., highlighting the complex interactions between the Wnt/β-catenin pathway activation and immune exclusion (20).

On the other hand, GC excess has been demonstrated to be strongly associated with depletion of T cell subtypes in ACC, both at the mRNA (56) and protein expression levels (13). Here, we found that LEF-1 protein expression is associated with the presence of clinical GC excess. Hence, any association of Wnt/β-catenin pathway activation will be confounded by direct GC excess.

However, while we observed a direct association between CTNNB1 and CD3^+^ immune cells, LEF-1 was not linked to any of the immune cell subtypes but interestingly associated with impaired expression of antigen presentation in tumours suggesting modulation of immune recognition.

We observed a less favourable survival for patients with high levels of LEF-1 but not high CTNNB1 expression.

Based on TCGA data, we hypothesized CTNNB1 expression levels to have a significant impact on overall survival. While the clinical characteristics of our cohort are similar with those of the TCGA cohort, TCGA has studied mRNA expression whereas we used protein expression by IHC on FFPE tissue samples. It is likely that mRNA expression does not fully reflect pathway activation given the complexity of pathway regulation and signalling crosstalk. In colorectal cancer, breast cancer and hepatocellular cancer, the activation of the Wnt/β-catenin pathway leads to a worse overall survival as high levels of CTNNB1 promotes metastasis and epithelial-mesenchymal transition (EMT) (57).

In contrast to a study by Wu et al., we did not find any correlation between the expression levels of GR and the immune cell infiltration levels or overall survival of the ACC patients (37).

In our study, we employed IHC to analyse LEF-1 expression, similar to the approach used in their West China Hospital (WCH) cohort. However, it is important to acknowledge the differences between the two studies, including variations in cohort size, clinical characteristics, and group classification methods. While the WCH cohort defined “high” expression as the top quartile, our study utilized the median expression level as the threshold for classification.

We found a positive correlation between cortisol secretion and LEF-1 expression levels, supporting our hypothesis that the Wnt/β-catenin pathway contributes significantly to immune exclusion in ACC, potentially through the mechanism of cortisol excess. These results not only corroborate previous findings but also strengthen the rationale for targeting these pathways in future treatment strategies.

The main limitations of our study is the relatively small number of samples. This is related to the rarity of the disease where large cohorts of clinically annotated patients are difficult to accrue. Further, in the absence of methods to directly assess intra-tumoural steroid concentration, we classified the samples according their clinical features.

Collectively, our work shows that LEF-1 can be used as a routine biomarker of Wnt/β-catenin pathway activation. Activation is not directly related to T cell infiltration but GC secretion in ACC and might play a role in antigen presentation, which needs further investigation.

The potential of LEF-1 expression as a biomarker for Wnt/β-catenin pathway activation could aid in patient selection for treatment with Wnt/β-catenin inhibitors, such as DCR BCAT. The promising results observed by Ganesh et al., where combination therapy with CTLA-4 and PD -1 antibodies led to complete remission in the majority of mice with Wnt-driven tumours, further underscore the therapeutic potential of targeting this pathway in ACC (58).

## Data Availability

The datasets presented in this study can be found in online repositories. The names of the repository/repositories and accession number(s) can be found in the article (41).
