# Supplementary material for: Wnt/β-catenin pathway activation is associated with glucocorticoid secretion in adrenocortical carcinoma, but not directly with immune cell infiltration
Source: Front Endocrinol (Lausanne). 2025 Mar 10;16:1502117. doi: 10.3389/fendo.2025.1502117 (PMC11930824; doi:10.3389/fendo.2025.1502117)
Supplement: Supplementary file 1 [file DataSheet1.pdf]

***Table S1**: ACC Patients´ and tumour characteristics divided by H-scores of LEF-1, CTNNB1, GR and the lymphocyte infiltration (CD3, CD4, CD8, FoxP3).*

Parameter	Immunohistochemistry cohort
Patients n	59
**Age at diagnosis (range)**	47 (19-75)
**Sex**	
Female	37 (63 %)
Male	20 (34 %)
unknown	2 (3 %)
**ENSAT Stage (I-IV)**	
I	4 (7 %)
II	27 (46 %)
III	15 (25 %)
IV	11 (19 %)
unknown	2 (3 %)
**Hormone status**	
Inactive	14 (24 %)
GC	15 (26 %)
A	3 (5 %)
multiple	10 (17 %)
unknown	16 (28 %)
**R-status (ENSAT I-III)**	unknown
0	
1	
2	
**Ki-67 index n (%)**	
<10	17 (28 %)
≥10	29 (50 %)
unknown	13 (22 %)

Colon LEF-1

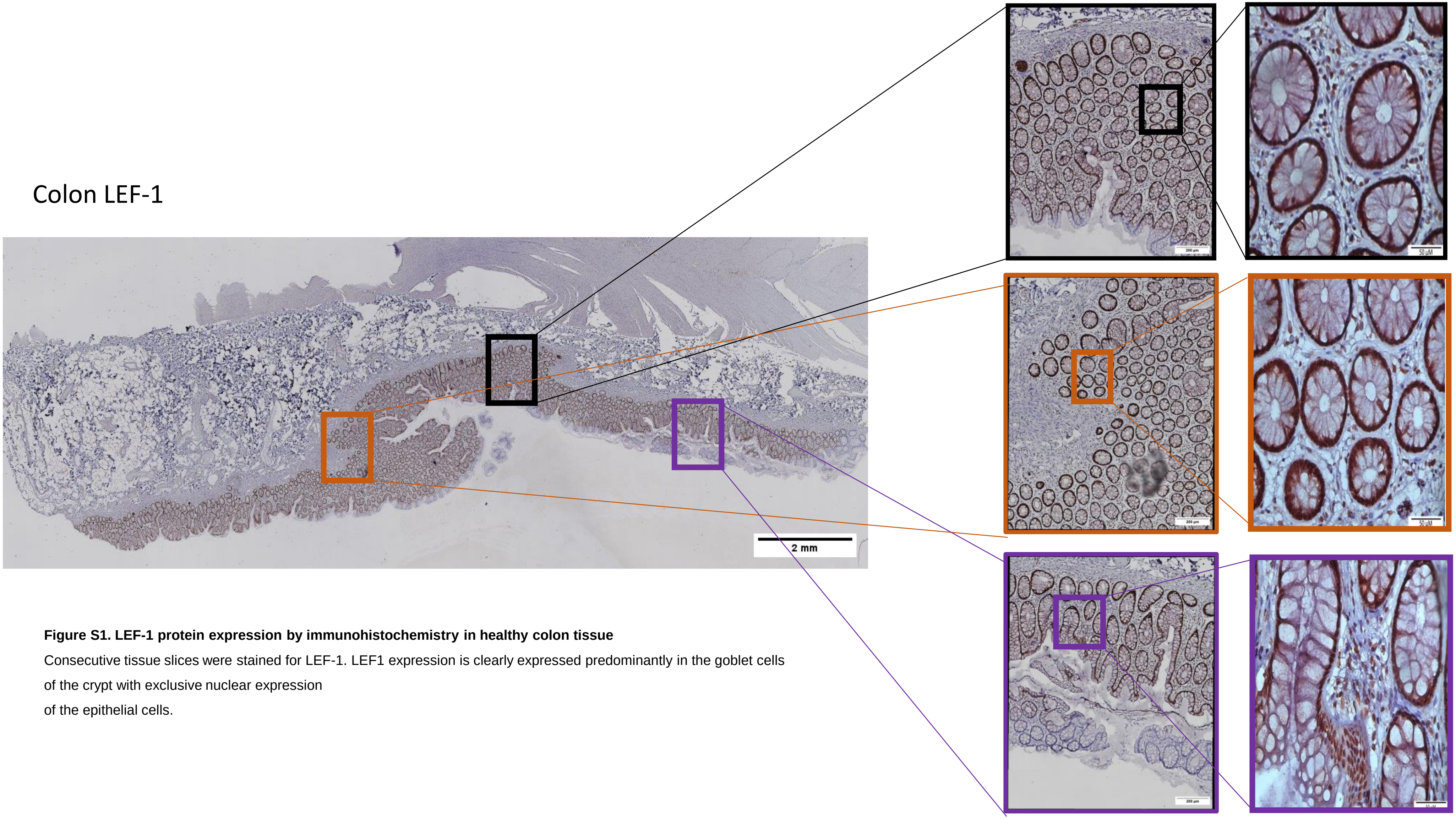

Figure S1. LEF-1 protein expression by immunohistochemistry in healthy colon tissue

Consecutive tissue slices were stained for LEF-1. LEF1 expression is clearly expressed predominantly in the goblet cells of the crypt with exclusive nuclear expression of the epithelial cells.

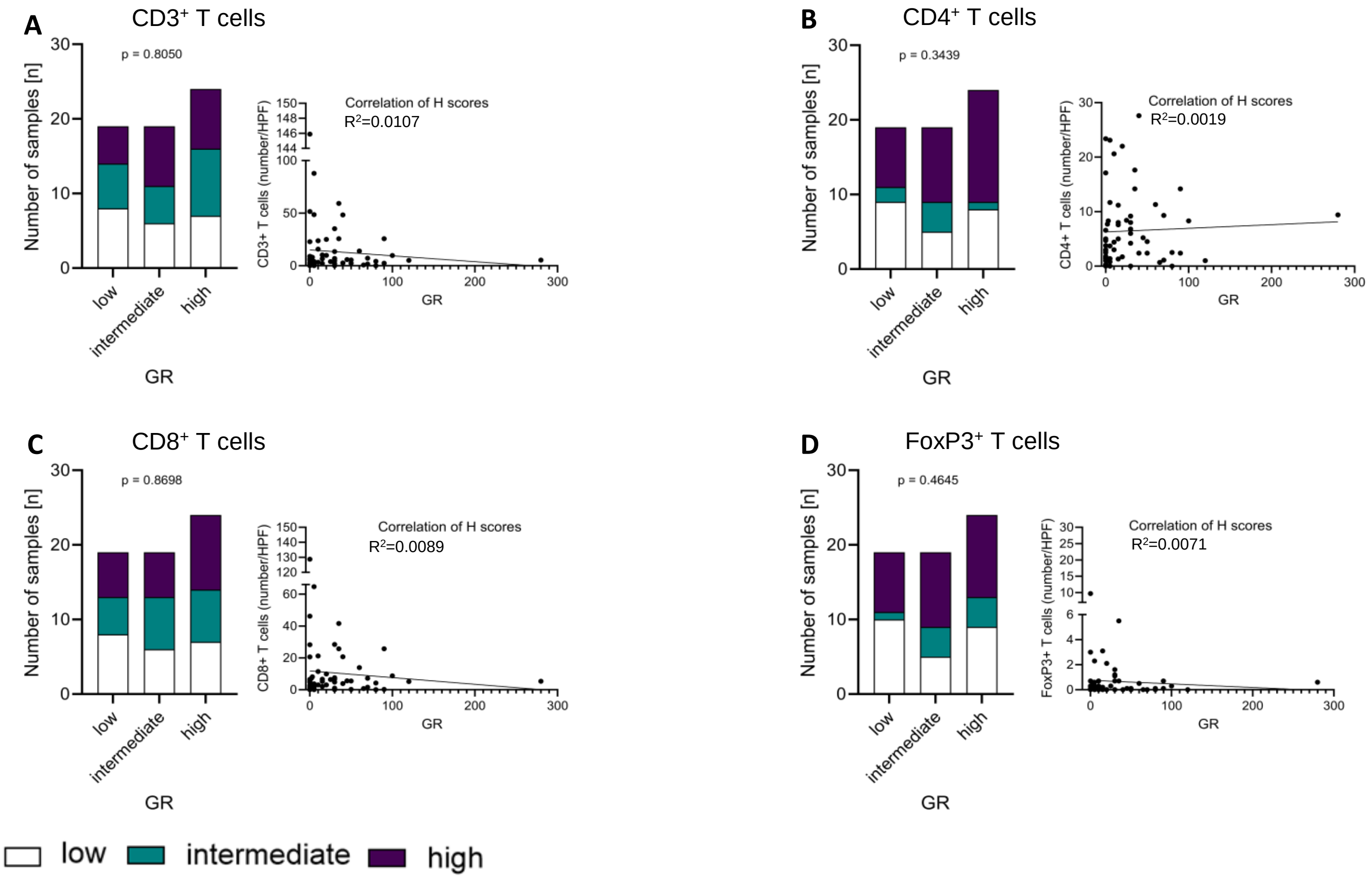

Figure S2, related to Figure 4 and 5. Correlation of GR and immune infiltration levels in the tumour. Protein expression levels of GR as well es CD3^{+} T cells (A), CD4^{+} T cells (B), CD8^{+} T cells (C), and FoxP3^{+} T cells (D) levels were divided into quartiles according to expression level and visualized in a stacked bar graph, respectively. The white colour is associated with low number of immune cells, the green is associated with intermediate number of immune cells and the lilac is associated with high levels of immune cells. Pearson correlation was used to show the linear relationship between the expression of GR (H-scores) and the immune cell infiltration (HPF), respectively. Testing for significance fisher´s exact test was used.

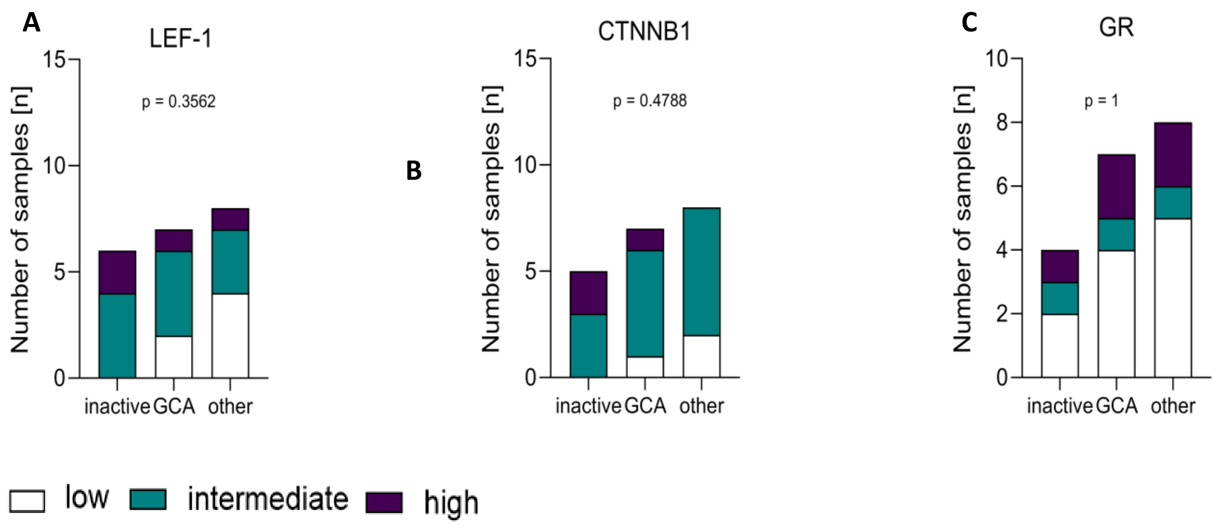

Figure S3, related to figure 6. Correlation of LEF-1 (A), CTNNB1 (B), and GR (C) with the hormone status, respectively. Hormone status of tumour (inactive, glucocorticoid excess (GC) or other (GCA, A, GCAO, multiple) were correlated and visualized as a stacked bar graph with the protein expression levels of LEF-1, CTNNB1 or GR, respectively. For statistical testing fisher´s exact test was used.

Table S2: *Numbers of T cell subtypes of CD3+, CD4+, CD8+, and FoxP3+ T cells and LEF-1 protein expression divided into tertiles .*

LEF-1 \ CD3	low	intermediate	high
low	7	6	8
intermediate	8	5	6
high	6	9	7

LEF-1 \ CD4	low	intermediate	high
low	8	5	8
intermediate	9	5	5
high	5	9	8

LEF-1 \ CD8	low	intermediate	high
low	7	7	7
intermediate	8	7	4
high	6	5	11

LEF-1 \ FoxP3	low	intermediate	high
low	9	2	10
intermediate	7	2	10
high	8	5	9

Table S3: *Numbers of T cell subtypes of CD3+, CD4+, CD8+, and FoxP3+ T cells and CTNNB1 protein expression divided into tertiles .*

CTNNB1 \ CD3	low	intermediate	high
low	6	2	13
intermediate	7	4	5
high	8	14	3

CTNNB1 \ CD4	low	intermediate	high
low	8	3	10
intermediate	6	4	6
high	8	12	5

CTNNB1 \ CD8	low	intermediate	high
low	6	5	10
intermediate	7	5	4
high	8	9	8

CTNNB1 \ FoxP3	low	intermediate	high
low	9	1	11
intermediate	5	0	11
high	10	8	7

Table S4: *Numbers of T cell subtypes of CD3+, CD4+, CD8+, and FoxP3+ T cells and CTNNB1 protein expression divided into tertiles.*

GR \ CD3	low	intermediate	high
low	8	6	5
intermediate	6	5	8
high	7	9	8

GR \ CD4	low	intermediate	high
low	9	2	8
intermediate	5	4	10
high	8	1	15

GR \ CD8	low	intermediate	high
low	8	5	6
intermediate	6	7	6
high	7	7	10

GR \ FoxP3	low	intermediate	high
low	10	1	8
intermediate	5	4	10
high	9	4	11

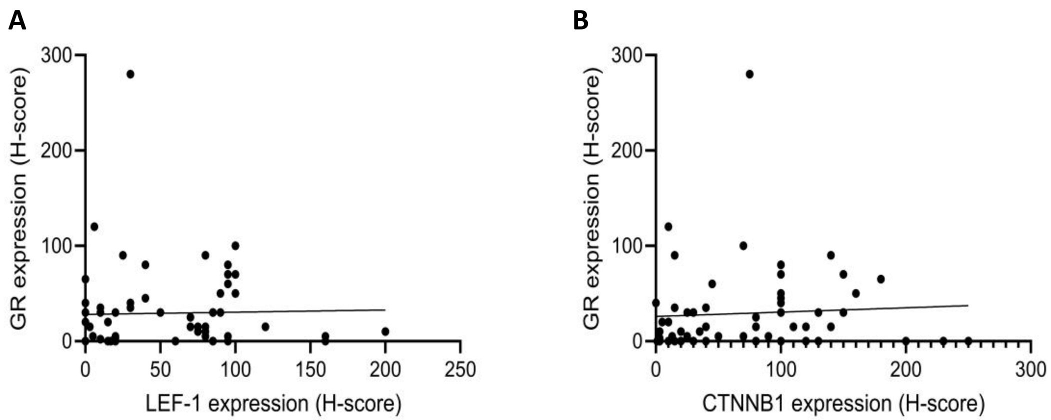

Figure S4. Correlation of GR expression (H-score) with LEF-1 and CTNNB1 H-score, respectively. GR expression (H-score) was correlated with the protein expression levels of LEF-1 expression (R^2=0.0006) and CTNNB1 (R^2=0.0040), respectively. For statistical testing simple linear regression was used.

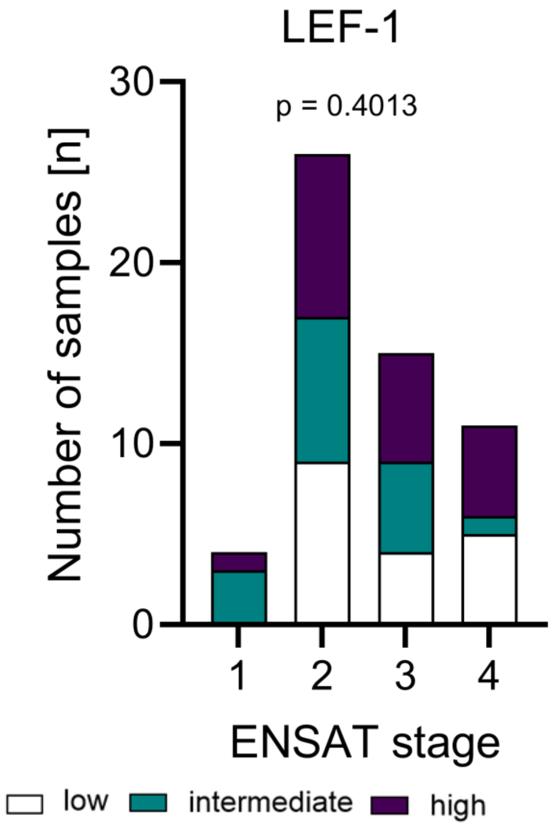

Figure S5. Correlation of ENSAT stage with LEF-1 (low, intermediate, high). ENSAT stage was correlated and visualized as a stacked bar graph with the protein expression levels of LEF-1 divided into low, intermediate and high. For statistical testing fisher´s exact test was used..

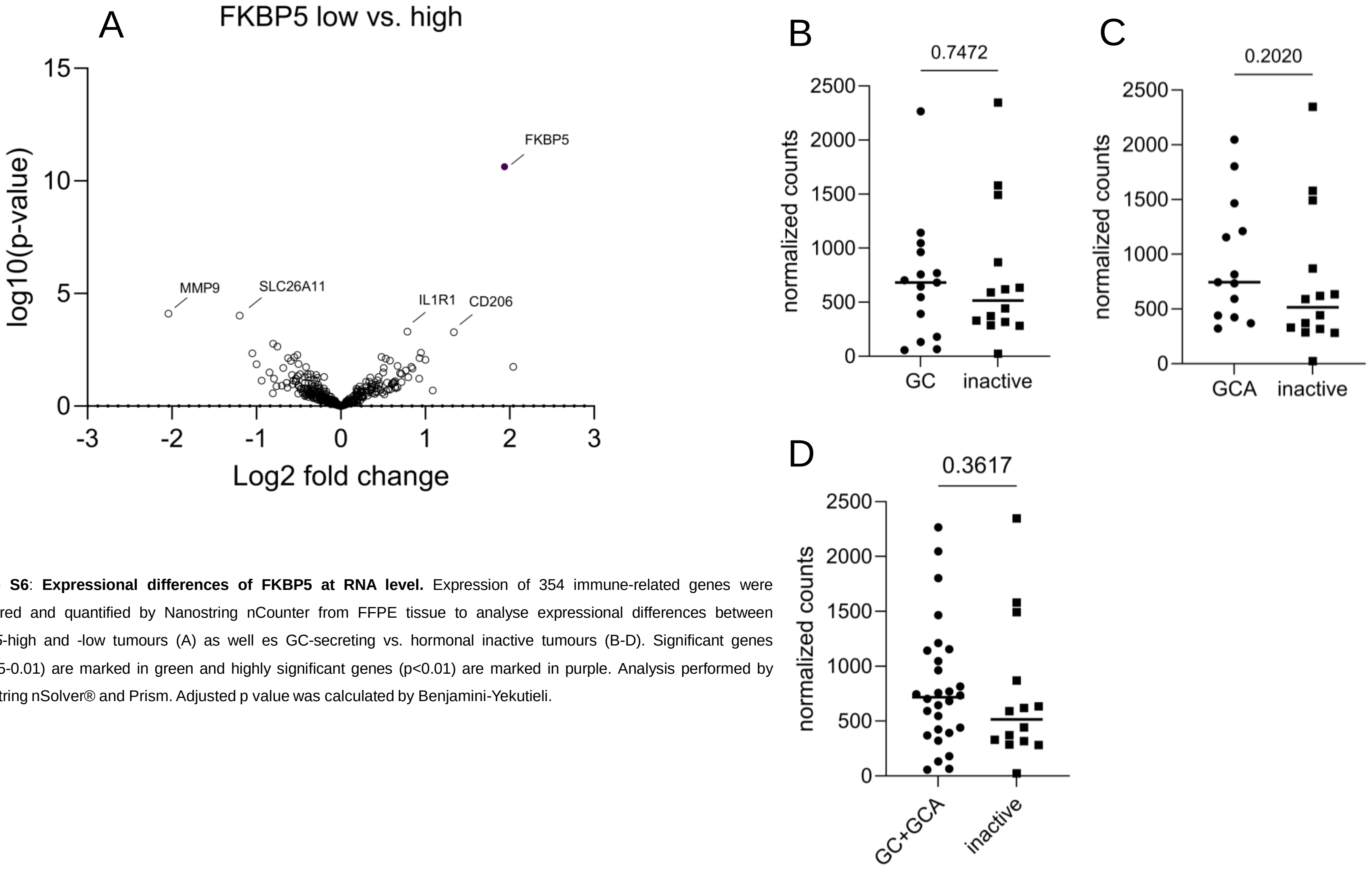

Figure S6: **Expressional differences of FKBP5 at RNA level.** Expression of 354 immune-related genes were measured and quantified by Nanostring nCounter from FFPE tissue to analyse expressional differences between *FKBP5*-high and -low tumours (A) as well es GC-secreting vs. hormonal inactive tumours (B-D). Significant genes (p<0.05-0.01) are marked in green and highly significant genes (p<0.01) are marked in purple. Analysis performed by Nanostring nSolver® and Prism. Adjusted p value was calculated by Benjamini-Yekutieli.
